# AI technostress and professional identity among Chinese university teachers: the mediating role of teaching self-efficacy and the moderating role of perceived organizational support

**DOI:** 10.3389/fpsyg.2026.1997877

**Published:** 2026-10-01

**Authors:** Youming Yang

**Affiliations:** Teachers College for Vocational and Technical Education, Guangxi Normal University, Guilin, Guangxi, China

**Keywords:** generative AI, job demands-resources model, perceived organizational support, professional identity, teaching self-efficacy, technostress, university teachers

## Abstract

Generative artificial intelligence (AI) has entered university teaching faster than the evidence needed to guide its use, and teachers carry much of the resulting strain. This study examined whether AI technostress is associated with the professional identity of university teachers through teaching self-efficacy, and whether perceived organizational support buffers this pathway. A cross-sectional questionnaire survey was conducted with a convenience sample of 486 full-time teachers from five public universities in Guangxi, China, all of whom had used at least one generative AI tool in their teaching or course preparation. Respondents completed Chinese adaptations of established measures of AI technostress, teaching self-efficacy, professional identity, and perceived organizational support. The measurement properties of the adapted scales were verified with confirmatory factor analysis and competing-model comparisons, and the hypotheses were tested with a moderated mediation path analysis based on 5,000 bootstrap resamples. AI technostress was negatively associated with professional identity, and teaching self-efficacy partially accounted for this association. Perceived organizational support moderated the first link of the pathway: the negative association between AI technostress and teaching self-efficacy was weaker among teachers who perceived stronger institutional backing, and the indirect association with professional identity was correspondingly smaller. These findings suggest that teachers’ professional identity is vulnerable to technology-related strain and that institutional support may attenuate this vulnerability. Universities that invest in efficacy-oriented professional development and visible organizational support are better positioned to help teachers sustain their professional identity as generative AI becomes embedded in teaching.

## 1 Introduction

Generative artificial intelligence (AI) has moved from a peripheral curiosity to a routine presence in higher education within a remarkably short period. Large language models such as ChatGPT and DeepSeek now assist with drafting, feedback, translation, and content generation, and universities across the world are weaving these tools into teaching, learning, and assessment (Kasneci et al., 2023; Lo, 2023; Tlili et al., 2023). Systematic reviews of artificial intelligence in higher education document rapid growth in applications alongside persistent gaps in pedagogical guidance and institutional policy (Holmes and Tuomi, 2022; Zawacki-Richter et al., 2019). The speed of this adoption has outrun the evidence base, leaving educators to make high-stakes decisions about when, how, and how much to rely on AI with little empirical grounding (Dwivedi et al., 2023). In China, where national strategies for educational digitalization encourage the integration of intelligent technologies into classrooms and AI policies in higher education are still being interpreted and enacted at the institutional level (Huang et al., 2026; Tsao, 2025), university teachers face concrete expectations to incorporate generative AI into their courses while institutional norms for such use remain fluid.

Much of the early research on generative AI in education has examined the learner side: student acceptance of ChatGPT, perceived benefits and risks, and the implications of chatbot use for academic integrity and learning quality (Chan and Hu, 2023; Chiu et al., 2023; Strzelecki, 2024). Teachers appear in this literature mainly as designers of AI-supported instruction or as adopters whose intentions are modeled with technology acceptance frameworks (Qadir, 2023). Qualitative work has begun to map university teachers’ beliefs about generative AI for teaching and the readiness concerns of beginning teachers (Cabellos et al., 2024; Moorhouse, 2024), and reviews of artificial intelligence in teacher professional development document rapidly growing applications with limited evidence on their consequences for teachers themselves (Tan et al., 2025). Considerably less attention has been paid to what sustained exposure to AI demands does to teachers’ professional selves.

The concept of technostress offers a productive entry point. Technostress describes the strain that arises when individuals cannot adapt to information technology in a healthy manner, and it is commonly decomposed into creators such as techno-overload, techno-invasion, techno-complexity, techno-insecurity, and techno-uncertainty (Ayyagari et al., 2011; Tarafdar et al., 2007). Meta-analytic evidence confirms that these creators relate consistently to strain and unfavorable job attitudes across work contexts (Kotek and Vranjes, 2025; Nastjuk et al., 2024), and related work has tied technostress to impaired concentration, sleep disturbance, and identity strain among intensive technology users (Salo et al., 2019). In higher education, Chinese university teachers reported lower teaching satisfaction and digital functioning under the demands of sudden technological change during the COVID-19 pivot to online teaching (Li and Yu, 2022), and studies of higher education teachers link perceived technostress to burnout and reduced wellbeing (Hogemann et al., 2025; Nascimento et al., 2024). Generative AI amplifies each creator: tools multiply faster than skills (complexity), update without warning (uncertainty), enter after-hours work (invasion), threaten established expertise (insecurity), and add to already heavy workloads (overload). What AI technostress means for how university teachers see themselves as professionals is the question pursued here.

The purpose of this study was to examine how AI technostress relates to the professional identity of Chinese university teachers, to test whether teaching self-efficacy accounts for part of this association, and to determine whether perceived organizational support conditions the strength of the link from technostress to self-efficacy. Figure 1 presents the conceptual model that guided the analysis.

**Figure 1:**
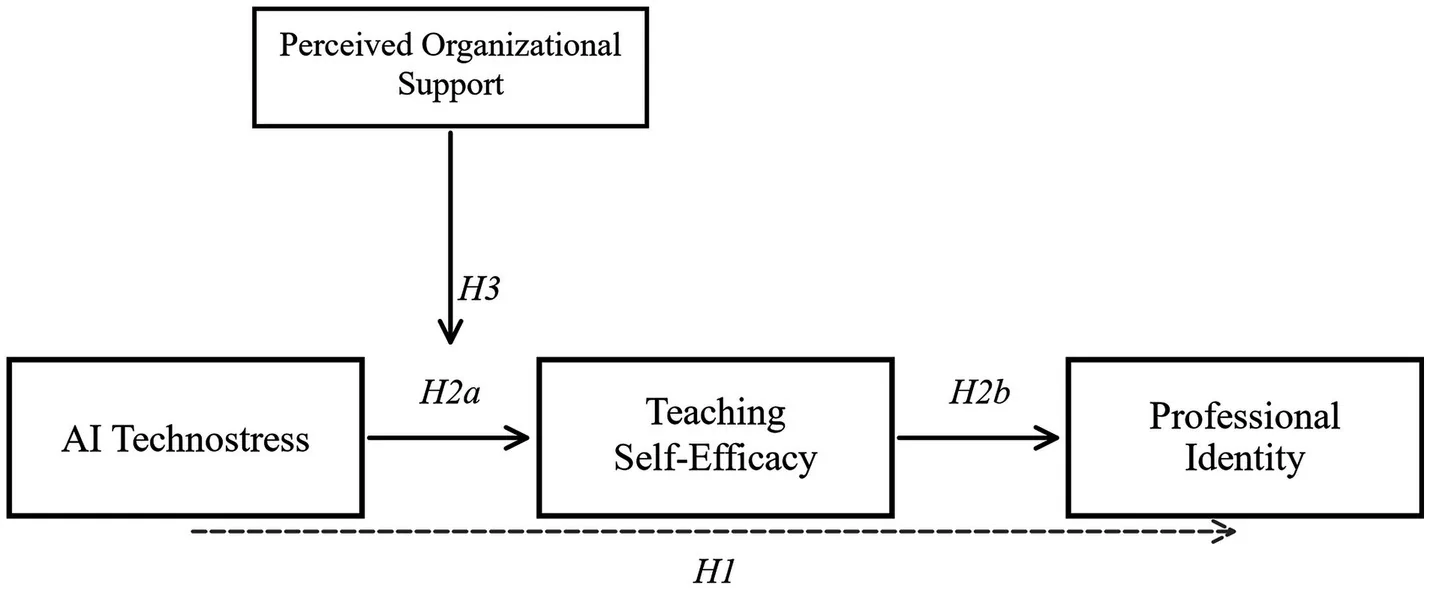
Conceptual model of the hypothesized moderated mediation. AI technostress is proposed to relate to professional identity directly (H1, dashed arrow) and indirectly through teaching self-efficacy (H2a and H2b, which jointly form the mediation hypothesis H2), and perceived organizational support moderates the path from AI technostress to teaching self-efficacy (H3).

The job demands-resources model provides the organizing framework. In this model, job demands consume energy and impair functioning, whereas job and personal resources buffer demands and sustain motivation and wellbeing (Bakker and Demerouti, 2007). Conservation of resources theory adds that individuals strive to protect valued resources, and that resource loss spirals when demands exceed the resources available to meet them (Hobfoll, 1989). AI technostress fits squarely on the demand side of this architecture. Among personal resources, self-efficacy occupies a central place: the belief in one’s capability to organize and execute courses of action shapes how demands are appraised and how much strain they produce (Bandura, 1993; Bandura, 1997). Teachers who feel efficacious in their teaching are better positioned to absorb new technological demands; teachers whose efficacy is strained by those demands are likely to experience the strain more acutely and to question their professional worth.

Professional identity captures the layer at which such questions are decided. Teachers’ professional identity, understood as the evolving answer to the question of who one is as a teacher, organizes commitment, motivation, and career decisions (Beijaard et al., 2004). Identity is dialogical and context-sensitive: it is continually negotiated between personal valuations and the expectations of the professional environment (Akkerman and Meijer, 2011). Contextualized measures show that a secure sense of professional identity covaries with occupational commitment and job satisfaction (Canrinus et al., 2011), and longitudinal evidence links a fragile identity to attrition from the teaching profession (Hong, 2010). Identity is also vulnerable to shifting policy and technological contexts, which can destabilize the values and practices around which teachers have built their professional selves (Beauchamp and Thomas, 2009; Day and Gu, 2007); interview evidence similarly shows that teachers actively renegotiate their professional identities amid the tensions that artificial intelligence introduces into their work (Lan, 2024). Generative AI poses precisely such a context shift: when machines draft feedback and generate instructional content, teachers may wonder which parts of their expertise still define them.

Teaching self-efficacy supplies the mechanism through which such destabilization may operate. Teaching self-efficacy, defined as a teacher’s belief in his or her capability to bring about desired student outcomes, predicts instructional quality, persistence, and resilience to stressors (Tschannen-Moran and Hoy, 2001). Efficacy beliefs are built and tested through experience: they develop across the early career and respond to the conditions under which teachers work (Hoy and Spero, 2005). Job stress depresses teacher self-efficacy across career stages (Klassen and Chiu, 2010), and low efficacy in turn accompanies burnout, a well-documented spiral in teacher samples (Skaalvik and Skaalvik, 2010). Chinese teacher samples show the same architecture, with self-efficacy and attributional patterns jointly predicting burnout and quitting intentions (Wang et al., 2015). Collective efficacy beliefs add a school-level layer to this system (Goddard et al., 2000), but the individual pathway remains the primary channel through which demands reach professional outcomes; recent scale development likewise confirms that efficacy beliefs regarding AI-supported teaching form a measurable and distinct construct (Bircan et al., 2026). When AI technostress chips away at a teacher’s sense of instructional competence, the loss is unlikely to stop at task confidence; it should reach the deeper question of what kind of professional one is. If AI technostress lowers teaching self-efficacy, and self-efficacy anchors professional identity, then technostress should relate to professional identity partly through efficacy, a mediation hypothesis that has not been tested in the generative AI context.

Whether this chain runs at full strength should depend on the organizational environment. Perceived organizational support, the extent to which employees believe their organization values their contribution and cares about their wellbeing, is among the most consistently documented job resources (Eisenberger et al., 1986). Meta-analytic evidence ties perceived support to favorable treatment, reduced strain, and stronger identification with the organization (Rhoades and Eisenberger, 2002), and identification processes link organizational membership to self-definition more broadly (Ashforth et al., 2008; Mael and Ashforth, 1992). Support theory implies a buffering role: when the university provides training, recognition, and workable policies for AI use, the same level of AI technostress should do less damage to teaching self-efficacy, because teachers appraise the demand as shared and manageable rather than as a private burden. Autonomy-supportive environments similarly protect motivational resources under pressure (Ryan and Deci, 2000). In job demands-resources terms, perceived organizational support is the resource most directly positioned to buffer the loss spiral from AI demands to efficacy strain.

The present study tested this moderated mediation model in a sample of Chinese university teachers. Four hypotheses were formulated. First, AI technostress would negatively predict professional identity (H1). Second, teaching self-efficacy would mediate this association, such that AI technostress predicts lower teaching self-efficacy, which in turn predicts weaker professional identity (H2). Third, perceived organizational support would moderate the path from AI technostress to teaching self-efficacy, with the negative association attenuated under high support (H3). Fourth, the indirect effect of AI technostress on professional identity through teaching self-efficacy would be conditional on perceived organizational support, yielding a significant index of moderated mediation (H4). The model connects the technostress literature with teacher identity research and speaks directly to calls for evidence-based guidance on teaching practice in the generative AI era.

## 2 Methods

### 2.1 Participants

A convenience sample of university teachers was recruited from five public universities in the Guangxi Zhuang Autonomous Region, China, located in Guilin, Nanning, and Liuzhou. Eligible respondents were full-time teaching staff who had used at least one generative AI tool in their teaching or course preparation during the current academic year. Questionnaires were distributed to 560 teachers, 512 were returned, and 26 were removed because of missing responses on more than 10% of items or obvious patterned responding, leaving 486 valid cases (94.9% of returned questionnaires). The final sample included 486 teachers (55.1% female) with a mean age of 37.2 years (SD = 7.2) and a mean teaching experience of 10.3 years. By academic rank, 18.1% were teaching assistants, 40.9% lecturers, 29.0% associate professors, and 11.9% full professors. Disciplinary backgrounds covered humanities (27.6%), social sciences (21.0%), natural sciences (18.7%), engineering (20.4%), and other fields (12.3%). Respondents reported moderate generative AI use for teaching on average (*M* = 3.01 on a five-point frequency scale), and 39.5% had attended at least one institutional training session on generative AI in education.

The target population was full-time teaching staff at public universities in the Guangxi Zhuang Autonomous Region, and the five participating universities were selected to vary in institutional mission and profile across three cities, with invitation links distributed through departmental teaching offices. The achieved sample size of 486 satisfied two planning criteria. With 47 observed indicators, the sample exceeded the commonly recommended ratio of 10 cases per indicator for confirmatory factor analysis. In addition, a sensitivity analysis for bivariate correlations indicated that a sample of this size has 80% power to detect correlations of *r* = 0.127 and 90% power to detect *r* = 0.147 (alpha = 0.05, two-tailed), so the design was sensitive to effects at least as small as those observed. As participation was voluntary, teachers who were more engaged with educational technology may have been somewhat overrepresented; this point is considered further in the limitations.

### 2.2 Measures

Unless noted otherwise, all items were rated on a five-point scale from 1 (strongly disagree) to 5 (strongly agree), and items within each scale were averaged to form composite scores. Original English items were translated into Chinese and back translated by two bilingual lecturers in educational technology following standard back-translation procedure (Brislin, 1970); discrepancies were resolved through discussion, and the questionnaire was piloted with 35 teachers from the same population, after which no item required substantive revision. Five bilingual experts in educational technology and educational measurement then rated the relevance of each adapted item to its intended construct; all items achieved item-level content validity indices of at least 0.80, and scale-level indices ranged from 0.90 to 0.96. The full set of 47 adapted items, the subscale each item belongs to, and the source it was adapted from are provided in Supplementary Table S1.

AI technostress was measured with 15 items adapted from the technostress creators framework (Ayyagari et al., 2011; Tarafdar et al., 2007) and its higher education operationalization (Wang et al., 2020), reworded to refer to generative AI use in teaching. Three items each covered techno-overload, techno-invasion, techno-complexity, techno-insecurity, and techno-uncertainty. A sample item is “I feel that generative AI tools force me to work with tighter time pressure than before.” Cronbach alpha was 0.899 in this sample.

Teaching self-efficacy was measured with 12 items adapted from the short form of the Teachers’ Sense of Efficacy Scale (Tschannen-Moran and Hoy, 2001), covering efficacy for instructional strategies, classroom management, and student engagement in courses that involve generative AI. A sample item is “I can craft good learning activities for my students even when generative AI tools are part of the task.” Cronbach alpha was 0.874.

Professional identity was measured with 12 items adapted from contextualized measures of teachers’ professional identity (Canrinus et al., 2011; Hong, 2010), covering occupational commitment, professional value, self-efficacious belonging, and volitional persistence in the teaching profession. A sample item is “Being a university teacher is an important part of who I am.” Cronbach alpha was 0.909.

Perceived organizational support was measured with eight items adapted from the short form of the Survey of Perceived Organizational Support (Eisenberger et al., 1986; Rhoades and Eisenberger, 2002), reworded to reference the university’s handling of generative AI in teaching. A sample item is “My university genuinely cares about the difficulties teachers face when using generative AI in their courses.” Cronbach alpha was 0.864.

Respondents also reported gender, age, years of teaching experience, academic rank, disciplinary background, frequency of generative AI use for teaching on a five-point scale from never to daily, and whether they had attended institutional training on generative AI in education.

### 2.3 Procedure and ethics

Data were collected online over 4 weeks in March and April 2026 through a secure survey platform. Departmental administrators at the participating universities forwarded the survey link to teaching staff; no administrator had access to individual responses. The first page of the questionnaire explained the purpose of the study, emphasized that participation was voluntary and anonymous, and stated that submission of the questionnaire constituted informed consent. No payment or course-related incentive was offered, and the questionnaire did not collect names or institutional identifiers. The study was approved by the Academic Ethics Committee of Guangxi Normal University and was conducted in accordance with local legislation and institutional requirements.

### 2.4 Data analysis

Preliminary analyses and confirmatory factor analysis (CFA) were conducted in Python (semopy 2.4). Model fit was evaluated with the chi-square test, CFI, TLI, RMSEA, and SRMR, applying conventional cutoffs (Hu and Bentler, 1999). The hypothesized four-factor measurement model was compared against a single-factor model, a second-order model in which the sub-facets of each measure loaded onto their intended higher-order constructs, and a four-factor model extended with an orthogonal common method factor loading on all items. Convergent validity was assessed with composite reliability, McDonald’s omega computed from the standardized loadings, and average variance extracted; discriminant validity was examined with the Fornell–Larcker criterion (Fornell and Larcker, 1981). Common method variance was examined with a Harman single-factor test (Podsakoff et al., 2003) and, more directly, with the latent common method factor model described above (Podsakoff et al., 2024). The hypothesized model was estimated as a path analysis on composite scores with mean-centered predictors and a product term for the moderation hypothesis; this specification is equivalent to a latent-score path analysis and was preferred because the measurement model had already established the psychometric properties of the four constructs. The indirect effect of AI technostress on professional identity through teaching self-efficacy was tested with 5,000 bootstrap resamples and percentile 95% confidence intervals, which do not assume normality of the sampling distribution of the indirect effect. Conditional indirect effects were evaluated at one standard deviation above and below the mean of perceived organizational support, and the index of moderated mediation was computed as the product of the interaction coefficient and the path from teaching self-efficacy to professional identity. Following the estimand logic of conditional process analysis (Hayes, 2022), the total effect was estimated in a model without the mediator and the moderator, whereas the direct effect and the conditional indirect effects were estimated in the full conditional process model; under a first-stage moderation these components are defined on different statistical models and need not sum to the total effect. Standardized coefficients are reported in the text and in Table 1, and the corresponding unstandardized coefficients, bootstrap confidence intervals, and simple slopes are reported in Table 2. Multicollinearity was examined with variance inflation factors. The significance level was set at 0.05 for all tests.

**Table 1:** Path model results: standardized coefficients and conditional indirect effects.

Path/effect	*β*	SE	*t*	*p*	95% CI
AI technostress → Teaching self-efficacy	−0.336	0.039	−8.59	<0.001	
POS → Teaching self-efficacy	0.310	0.039	7.93	<0.001	
AI technostress × POS → Teaching self-efficacy	0.216	0.039	5.59	<0.001	
AI technostress → Professional identity (direct)	−0.350	0.040	−8.81	<0.001	
Teaching self-efficacy → Professional identity	0.240	0.042	5.74	<0.001	
POS → Professional identity	0.217	0.039	5.52	<0.001	
Indirect effect at mean POS	−0.084				[−0.120, −0.052]
Conditional indirect at low POS (−1 SD)	−0.137				[−0.191, −0.087]
Conditional indirect at high POS (+1 SD)	−0.030				[−0.062, −0.002]
Index of moderated mediation	0.062				[0.035, 0.092]
Total effect (AI technostress → Professional identity)	−0.497	0.039	−12.59	<0.001	

*N* = 486. *β*, standardized coefficient; predictors were mean-centered and the product term was computed from centered variables. *R*^2^ = 0.300 for teaching self-efficacy and 0.370 for professional identity. The total effect was estimated in a model without the mediator and the moderator; all other coefficients come from the full conditional process model. Unstandardized coefficients for all paths and effects are given in Table 2. Indirect effects were tested with 5,000 bootstrap resamples and percentile confidence intervals. POS, perceived organizational support.

**Table 2:** Unstandardized path coefficients, bootstrap confidence intervals, and simple slopes.

Path/effect	*B*	SE	*t*	*p*	95% CI
AI technostress → Teaching self-efficacy	−0.338	0.039	−8.59	<0.001	
POS → Teaching self-efficacy	0.280	0.035	7.93	<0.001	
AI technostress × POS → Teaching self-efficacy	0.251	0.045	5.59	<0.001	
Teaching self-efficacy → Professional identity	0.248	0.043	5.74	<0.001	
AI technostress → Professional identity (direct)	−0.362	0.041	−8.81	<0.001	
POS → Professional identity	0.202	0.037	5.52	<0.001	
Total effect (model without *M* and moderator)	−0.515	0.041	−12.59	<0.001	
Indirect effect at mean POS	−0.084				[−0.120, −0.053]
Conditional indirect at low POS (−1 SD)	−0.137				[−0.193, −0.089]
Conditional indirect at high POS (+1 SD)	−0.030				[−0.062, −0.003]
Index of moderated mediation	0.062				[0.036, 0.092]
Simple slope: TS → SE at low POS (−1 SD)	−0.555				
Simple slope: TS → SE at mean POS	−0.338				
Simple slope: TS → SE at high POS (+1 SD)	−0.120				

*N* = 486. *B*, unstandardized coefficient; predictors were mean-centered. The total effect was estimated in a model without the mediator and the moderator; all other coefficients come from the full conditional process model. Indirect effects, the index of moderated mediation, and simple slopes are unstandardized; confidence intervals are percentile bootstrap intervals based on 5,000 resamples. TS, AI technostress; SE, teaching self-efficacy; POS, perceived organizational support.

## 3 Results

### 3.1 Preliminary analyses

Table 3 presents descriptive statistics and correlations. All four scales showed good internal consistency (alphas from 0.864 to 0.909). AI technostress correlated negatively with teaching self-efficacy (*r* = −0.407), professional identity (*r* = −0.497), and perceived organizational support (*r* = −0.226). Teaching self-efficacy correlated positively with professional identity (*r* = 0.466) and perceived organizational support (*r* = 0.382), and support correlated positively with identity (*r* = 0.388). The pattern and magnitude of these correlations are in line with recent teacher and employee samples linking technostress to strain and reduced job attitudes (Nascimento et al., 2024; Nastjuk et al., 2024). A single factor extracted in an unrotated exploratory analysis accounted for 25.7% of the total variance, well below the 50% threshold, suggesting that common method variance was not a serious concern.

**Table 3:** Descriptive statistics and correlations among study variables (*N* = 486).

Variable	Items	*M*	SD	*α*	1	2	3
1. AI technostress	15	3.05	0.78	0.899	–		
2. Teaching self-efficacy	12	3.62	0.78	0.874	−0.407	–	
3. Professional identity	12	3.71	0.81	0.909	−0.497	0.466	–
4. Perceived organizational support	8	3.33	0.87	0.864	−0.226	0.382	0.388

*α*, Cronbach alpha. Correlations with perceived organizational support: *r* = −0.226 with AI technostress, *r* = 0.382 with teaching self-efficacy, *r* = 0.388 with professional identity. All correlations are significant at *p* < 0.001.

### 3.2 Measurement model

The four-factor CFA showed good fit: chi-square (1,028) = 1,161.8 (chi-square/df = 1.13), CFI = 0.984, TLI = 0.983, RMSEA = 0.016, SRMR = 0.035. The chi-square test was significant, as is common with samples of several hundred cases, so we relied primarily on the incremental and absolute fit indices. Standardized loadings ranged from 0.54 to 0.73, and all were significant at *p* < 0.001. As shown in Table 4, composite reliability exceeded 0.85 for every construct. Reliability was additionally computed as McDonald’s omega from the standardized loadings; the omega values were nearly identical to the Cronbach alpha and composite reliability values for every scale, a convergence that reflects the high homogeneity of the standardized loadings within each measure. Average variance extracted ranged from 0.368 to 0.455, below the 0.50 benchmark that is often difficult to reach for scales aggregating heterogeneous facets. Following the Fornell–Larcker criterion (Fornell and Larcker, 1981), the square root of each average variance extracted exceeded the corresponding inter-construct correlations for every pair of constructs, supporting discriminant validity, and convergent validity was regarded as acceptable given the reliability of the composites.

**Table 4:** Reliability and convergent validity of the measurement model.

Scale	Cronbach *α*	*ω*	CR	AVE	Loading range
AI technostress	0.899	0.899	0.899	0.373	0.55–0.64
Teaching self-efficacy	0.874	0.874	0.874	0.368	0.54–0.66
Professional identity	0.909	0.909	0.909	0.455	0.62–0.73
Perceived organizational support	0.864	0.864	0.864	0.443	0.62–0.70

*N* = 486. Fit of the four-factor model: *χ*^2^ (1,028) = 1,161.8, CFI = 0.984, TLI = 0.983, RMSEA = 0.016, SRMR = 0.035. All loadings were significant at *p* < 0.001. *ω*, McDonald’s omega computed from the standardized CFA loadings; CR, composite reliability; AVE, average variance extracted. The proximity of *ω*, *α*, and CR reflects the high homogeneity of the standardized loadings within each scale. The square root of each AVE exceeded the corresponding inter-construct correlations for every pair of constructs (Fornell–Larcker criterion).

The hypothesized four-factor model was compared against three alternatives (Table 5). A single-factor model fit the data poorly (CFI = 0.616, RMSEA = 0.079, SRMR = 0.096). A second-order model in which the sub-facets of each measure loaded onto their intended higher-order constructs fit as well as the four-factor model, indicating that the composites summarize coherent facet structures. A four-factor model extended with an orthogonal common method factor improved fit only trivially: the mean absolute standardized loading on the method factor was 0.122, and the change in CFI relative to the four-factor model was 0.0035, below the 0.01 criterion, suggesting that common method variance did not substantively distort the measurement (Podsakoff et al., 2024). Together with the Harman single-factor result, these analyses indicate that the observed associations are unlikely to be artifacts of the single-source design.

**Table 5:** Comparison of competing measurement models.

Model	*χ* ^2^	df	*χ*^2^/df	CFI	TLI	RMSEA	SRMR
Four-factor model	1,161.8	1,028	1.130	0.984	0.983	0.016	0.035
Single-factor model	4,161.4	1,034	4.025	0.616	0.598	0.079	0.096
Second-order model	1,153.7	1,016	1.136	0.983	0.982	0.017	0.034
Four-factor + common method factor	1,086.4	981	1.107	0.987	0.986	0.015	0.033

*N* = 486. The common method factor was specified as orthogonal to the four constructs and loaded on all 47 items. The change in CFI relative to the four-factor model was 0.0035, below the 0.01 criterion for a meaningful method-factor improvement. The second-order model specified the sub-facets of each measure as first-order factors loading on their intended higher-order constructs.

### 3.3 Hypothesis testing

The path model is summarized in Table 1. Hypothesis 1 was supported: the total association between AI technostress and professional identity was negative and significant (beta = −0.497, *p* < 0.001), and the direct association remained significant when teaching self-efficacy and perceived organizational support were controlled (beta = −0.350, *p* < 0.001). Supporting H2, AI technostress negatively predicted teaching self-efficacy (beta = −0.336, *p* < 0.001), teaching self-efficacy positively predicted professional identity (beta = 0.240, *p* < 0.001), and the indirect effect through teaching self-efficacy was −0.084 (95% CI [−0.120, −0.052]), accounting for 16.3% of the total association. The model explained 30.0% of the variance in teaching self-efficacy and 37.0% of the variance in professional identity.

Consistent with the estimation plan, the total effect was derived from a model without the mediator and the moderator, whereas the direct effect and the conditional indirect effects were derived from the full conditional process model; under a first-stage moderation these components are defined on different statistical models and need not sum to the total effect (Hayes, 2022). Variance inflation factors ranged from 1.00 to 1.34 across the predictors of both equations, indicating no multicollinearity concern. The corresponding unstandardized coefficients, bootstrap confidence intervals, and simple slopes are reported in Table 2.

Hypotheses 3 and 4 also received support. The interaction between AI technostress and perceived organizational support predicted teaching self-efficacy (beta = 0.216, *p* < 0.001): the negative association between technostress and self-efficacy was stronger when support was low and weaker when support was high, consistent with a buffering account. Perceived organizational support also showed direct positive associations with teaching self-efficacy (beta = 0.310, *p* < 0.001) and professional identity (beta = 0.217, *p* < 0.001). The conditional indirect effect of AI technostress on professional identity through teaching self-efficacy was −0.137 (95% CI [−0.191, −0.087]) at low perceived support (one standard deviation below the mean) and −0.030 (95% CI [−0.062, −0.002]) at high perceived support (one standard deviation above the mean); both intervals excluded zero, but the effect at low support was more than four times the effect at high support. The index of moderated mediation was 0.062 (95% CI [0.035, 0.092]), confirming that the indirect pathway varied significantly with perceived organizational support. Simple slope analyses summarized in Table 2 showed that the unstandardized association between AI technostress and teaching self-efficacy was strongest at low support (*B* = −0.555), intermediate at average support (*B* = −0.338), and weakest at high support (*B* = −0.120); the interaction pattern is depicted in Figure 2.

**Figure 2:**
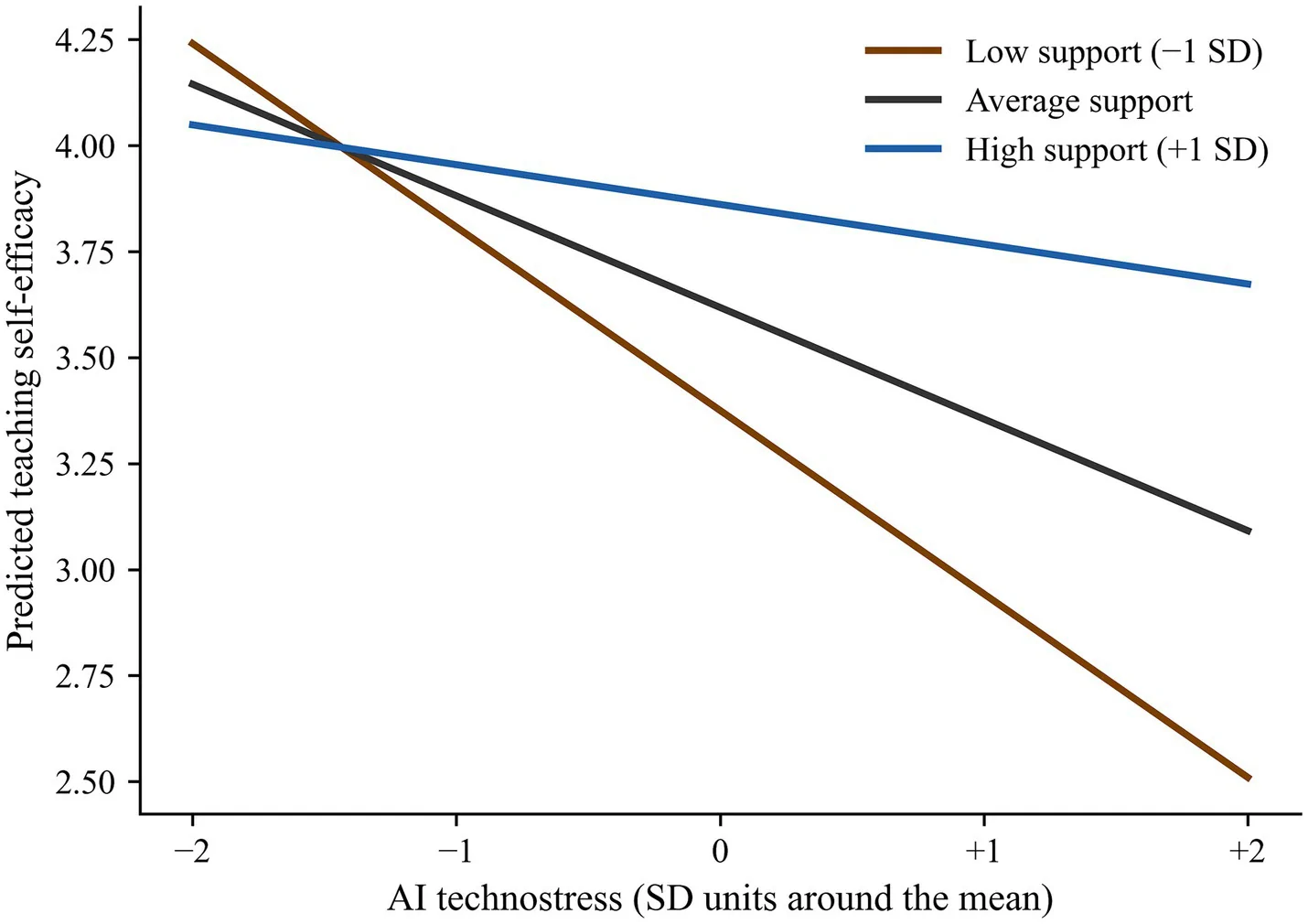
Two-way interaction between AI technostress and perceived organizational support in predicting teaching self-efficacy. Lines represent simple slopes at one standard deviation below the mean, at the mean, and at one standard deviation above the mean of perceived organizational support.

## 4 Discussion

This study asked how AI technostress relates to the professional identity of university teachers and what machinery connects the two. Among 486 teachers from five public universities in Guangxi, AI technostress was associated with weaker professional identity both directly and through teaching self-efficacy: teachers who reported heavier AI-related strain also reported lower confidence in their teaching, and this lower confidence accompanied a weaker sense of professional identity. Perceived organizational support was associated with a weaker first link of this chain: the negative association between technostress and teaching self-efficacy was attenuated at high support, and the indirect effect on identity shrank to less than a quarter of its magnitude under low support.

The negative association between AI technostress and professional identity is the central finding. Teachers who reported heavier AI-related strain reported a weaker sense of professional self, consistent with identity theory, which treats the professional self as continuously negotiated between personal valuations and contextual demands (Akkerman and Meijer, 2011; Beijaard et al., 2004) and with recent evidence that teachers actively renegotiate their professional identities amid AI-induced tensions (Lan, 2024). The mediation finding positions teaching self-efficacy as a transmission belt between technological demands and professional self-definition. Prior work established that job stress depresses teacher self-efficacy (Klassen and Chiu, 2010) and that low efficacy accompanies maladaptive outcomes such as burnout (Skaalvik and Skaalvik, 2010; Wang et al., 2015). The present results extend this architecture to a new class of demand. Generative AI challenges the sources from which efficacy beliefs are built: mastery experiences become less stable when tools change monthly, and vicarious comparison is complicated when AI can simulate competent performance (Bandura, 1993). The co-occurrence of lower efficacy and weaker identity in our model fits this reading: teachers who no longer feel capable of directing learning in AI-saturated classrooms appear to reassess not only what they can do but who they are professionally.

The moderation finding identifies perceived organizational support as a boundary condition of this process. The buffering pattern matches the logic of organizational support theory, under which valued and cared-for employees appraise demands as shared obligations rather than private threats (Eisenberger et al., 1986; Rhoades and Eisenberger, 2002). It also fits conservation of resources theory: institutional backing replenishes the resource pool that technostress depletes, slowing the loss spiral before it reaches efficacy beliefs (Hobfoll, 1989). Support also showed direct positive associations with both efficacy and identity, which suggests that it operates additively as well as conditionally, a dual role documented for organizational resources in teacher samples more generally (Day and Gu, 2007). In practical terms, the conditional indirect effect under high support was small, suggesting that the identity-relevant association of AI strain was substantially weaker among well-supported teachers even when the strain itself was present.

For universities navigating generative AI integration, three recommendations follow from these findings. First, professional development should target teaching self-efficacy rather than tool familiarity alone. Because the association between AI technostress and identity operated partly through teaching self-efficacy, training that builds genuine mastery experiences with AI tools, such as guided redesign of one’s own course tasks with coaching and peer observation, is more likely to support both efficacy and identity than pure tool demonstrations (Hoy and Spero, 2005; Tschannen-Moran and Hoy, 2001). Second, institutional support should be visible and specific. Workable policies on AI use, recognition of the extra labor that AI-integrated teaching requires, and channels through which teachers can report difficulties all signal organizational care, and the moderation results indicate that such signals were associated with a weaker strain-to-efficacy link in this sample. Third, as generative-AI literacy frameworks for teachers mature (Bircan et al., 2026; Wang et al., 2025), universities would be well advised to embed technostress monitoring into routine faculty development rather than treating AI-related strain as an individual coping problem. None of these recommendations requires slowing AI adoption; all concern how the adoption is resourced and governed (Dwivedi et al., 2023; Holmes and Tuomi, 2022).

Several limitations should be weighed. The design was cross-sectional, so the model describes associations rather than causal effects, and reverse or reciprocal pathways between efficacy and identity remain plausible. All measures were self-reported; although the Harman test was reassuring, the competing-model comparisons supported the four-factor structure, and the latent method factor accounted for little additional variance, method variance cannot be excluded (Podsakoff et al., 2024). Respondents were volunteers recruited by convenience from one region of China, so teachers who are more engaged with educational technology may be overrepresented, and generalization to other regions, institutional types, and career stages requires caution. Perceptions were measured at a single point in the early institutionalization of generative AI and may shift as the technology matures and longitudinal adoption patterns unfold (Zhou et al., 2026). Since the questionnaire did not collect institutional identifiers, responses could not be clustered by university, so possible nested dependencies among teachers within institutions could not be modeled; multi-site designs that permit such modeling are needed. AI technostress was assessed as a global composite; future work could examine which creators, such as techno-insecurity or techno-uncertainty, carry the identity-relevant variance. Finally, the study measured perceptions of support rather than objective institutional provision, and the two may diverge. Longitudinal and experience-sampling designs could trace how technostress, efficacy, and identity co-develop as AI tools mature, and intervention studies could test whether efficacy-focused training helps sustain professional identity over time.

## 5 Conclusion

Among Chinese university teachers, AI technostress was associated with weaker professional identity partly through lower teaching self-efficacy, and perceived organizational support was associated with a substantially smaller conditional indirect effect under high support. The study connects technostress research with teacher identity scholarship and suggests that efficacy-focused training combined with visible institutional support may help teachers sustain their professional selves as generative AI becomes embedded in university teaching. These conclusions are correlational and await confirmation in longitudinal and intervention work.

## Data Availability

The raw data supporting the conclusions of this article will be made available by the authors, without undue reservation.
