# Supplementary material for: AI technostress and professional identity among Chinese university teachers: the mediating role of teaching self-efficacy and the moderating role of perceived organizational support
Source: Front Psychol. 2026 Oct 1;17:1997877. doi: 10.3389/fpsyg.2026.1997877 (PMC13668839; doi:10.3389/fpsyg.2026.1997877)
Supplement: Supplementary file 1 [file Data_Sheet_1.PDF]

Supplementary Material

AI Technostress and Professional Identity Among Chinese University Teachers: The Mediating Role of Teaching Self-Efficacy and the Moderating Role of Perceived Organizational Support

Table S1. Adapted item pool (47 items). Section headings give the scale and the sources from which its items were adapted; items were reworded to refer to generative AI use in university teaching. Items were translated into Chinese and back translated by two bilingual lecturers in educational technology, piloted with 35 teachers from the target population, and rated for relevance by five bilingual experts in educational technology and educational measurement; all items achieved item-level content validity indices of at least 0.80, and scale-level indices ranged from 0.90 to 0.96. The Chinese version was administered; the English back-translations are shown below. All items were rated from 1 (strongly disagree) to 5 (strongly agree).

No.	Item
**AI technostress (15 items; adapted from Tarafdar et al., 2007; Ayyagari et al., 2011; Wang et al., 2020)**	
Techno-overload	
1	I feel that generative AI tools force me to work with tighter time pressure than before.
2	I have to process more information because of generative AI tools than I used to.
3	Generative AI tools make me work faster than I am comfortable with.
Techno-invasion	
4	Work related to generative AI intrudes into my after-hours and weekend time.
5	Generative AI expectations keep me reachable for work matters beyond working hours.
6	Generative AI developments follow me into my private time.
Techno-complexity	
7	Generative AI tools are complex and difficult to understand and use.
8	I need a long time to learn how to use new generative AI tools properly.
9	I do not find enough time to learn and improve my generative AI skills.
Techno-insecurity	
10	I feel my job security could be threatened as generative AI takes over parts of my teaching tasks.
11	I worry that colleagues who are more skilled with generative AI may be valued more than I am.
12	I feel threatened that generative AI could replace some of my professional contributions.
Techno-uncertainty	
13	Generative AI tools change so quickly that I have trouble keeping up.
14	There are always new versions and functions of generative AI tools to learn.
15	I cannot predict which generative AI tools will be expected in my teaching in the future.
**Teaching self-efficacy (12 items; adapted from Tschannen-Moran and Hoy, 2001)**	
Efficacy for instructional strategies	
16	I can use a variety of instructional strategies in courses that involve generative AI.
17	I can provide an alternative explanation or example when students are confused about AI-related course content.
18	I can craft good learning activities for my students even when generative AI tools are part of the task.
19	I can adjust my instruction to the needs of different students in AI-supported courses.
Efficacy for classroom management	
20	I can control disruptive behavior in a class that uses generative AI tools.
21	I can make my expectations about appropriate AI use clear to students.
22	I can keep students on task in learning activities that involve generative AI.
23	I can address misuse of generative AI tools during class effectively.
Efficacy for student engagement	
24	I can motivate students who show low interest in learning with generative AI tools.
25	I can help my students value learning even when generative AI tools could provide quick answers.

26	I can get students to believe they can do well in courses that use generative AI.
27	I can engage students in critical thinking about the outputs of generative AI tools.
**Professional identity (12 items; adapted from Canrinus et al., 2011; Hong, 2010)**	
Occupational commitment	
28	Being a university teacher is an important part of who I am.
29	I am proud to tell others that I work as a university teacher.
30	I feel committed to remaining in the teaching profession.
Professional value	
31	I believe that university teaching is a profession of great social value.
32	I feel that my work as a teacher makes a meaningful contribution to society.
33	I value the professional standards associated with being a university teacher.
Self-efficacious belonging	
34	I feel that I belong to the community of university teachers.
35	I see myself as a typical university teacher.
36	I share the values that university teachers generally hold.
Volitional persistence	
37	If I had to choose again, I would still choose to become a university teacher.
38	I intend to stay in the teaching profession even when my work becomes demanding.
39	I cannot imagine doing work other than university teaching in the future.
**Perceived organizational support (8 items; adapted from Eisenberger et al., 1986; Rhoades and Eisenberger, 2002)**	
40	My university genuinely cares about the difficulties teachers face when using generative AI in their courses.
41	My university values the extra effort that AI-integrated teaching requires.
42	My university provides help when I encounter problems with generative AI tools in my teaching.
43	My university strongly considers my goals and values when making decisions about generative AI use.
44	My university shows concern for my well-being as generative AI changes my teaching work.
45	My university is willing to provide resources that help me teach with generative AI.
46	My university takes pride in my accomplishments with generative AI in my courses.
47	If I had difficulties with generative AI in my teaching, my university would try to help me.
